# Pressure sensing technology for remote control: Can we motivate users to stay on the learning curve?

**DOI:** 10.1371/journal.pone.0340667

**Published:** 2026-03-10

**Authors:** Emily M. Crowe, Simon Castle-Green, Daisy Beecroft, Praminda Caleb-Solly

**Affiliations:** 1 School of Psychology, University of Nottingham, Nottingham, United Kingdom; 2 School of Computer Science, University of Nottingham, Nottingham, United Kingdom; Montclair State University, UNITED STATES OF AMERICA

## Abstract

Learning to use a novel human-in-the-loop control system is often a slow and frustrating process due to the need to understand new interaction paradigms. Unsurprisingly, research has commonly focused on identifying methods to accelerate such learning. In this paper we consider an alternative approach of motivating learners to persist with their learning. If learners are motivated to continue investing time in using a novel control system they will transition to proficiency, albeit at different timescales. Participants controlled the movements of a virtual robot in real-time by adjusting their movements whilst seated on a pressure sensing mat. In two experiments, participants played a game where their task was to move a virtual robot to collect targets as quickly as possible. Targets were only presented for a fixed duration such that participants received binary reward feedback dependent upon whether they collected a given target in time or not. This feedback was used to calculate each participant’s success frequency which was used as a proxy for their skill level and thus learning. Experiment 1 showed that participants could learn the control system but that their motivation to play the game decreased as the experiment continued. Experiment 2 investigated whether adapting task difficulty as a function of the participants’ current skill level (indexed by success frequencies) would increase the time participants chose to invest playing the game. Participants did not choose to play the game for longer when playing the game under this adaptive difficulty condition compared with fixed difficulty conditions. We conclude that most participants improved at using the pressure sensing mat for remote control but adapting the difficulty of the task to participant’s skill level did not increase the time they were willing to invest in playing it.

## Introduction

Over the years human-computer interaction using computer mice and joysticks has evolved into more sophisticated remote control such as telerobotic surgery and deep-sea exploration. Proficient use of such technologies requires motor learning whereby users must learn the mapping between their movements and those of the tool or robot they are controlling remotely. This type of learning is often called ‘de novo learning’ because a new controller is learnt from scratch rather than adapted from a pre-existing controller [1–4]. De novo learning occurs over longer timescales than adaptation, often taking hours or days and thus learning skills like teleoperation require considerable time investment from a user [5,6]. This is thought to be due to a combination of factors including the use of somewhat arbitrary mappings and the need to generate unfamiliar actions consistently [7]. Unsurprisingly, there is a growing body of research seeking to identify methods for accelerating the learning of novel control systems.

The different approaches for accelerating learning of a novel control system can be classified according to which component of the human-computer interaction they aim to improve. One approach is to optimize the computer or robot’s performance by, for example, using sonar sensors for collision avoidance in robots [e.g., 8]. An alternative approach is to focus on optimizing human performance by, for example, relaying sensory feedback to the human in a way that considers the limits of their sensorimotor system (e.g., difficulties with depth perception) [9–13]. These approaches have been successful but are susceptible to individual differences, such as the users’ ability to embody a robotic system [14] and expectations regarding how to interpret sensory feedback [15].

An alternative approach for facilitating the learning of novel control systems is to find methods to motivate users to persist with their learning, rather than accelerating it. Broadly, motivation describes the drive to complete a task and achieve one’s goals [16]. It is multifaceted concept that plays a role in people’s abilities to learn new motor skills [17,18] such as those required to successfully use novel technologies like using a trackpad to control a cursor on a computer screen or one’s finger to swipe to read an article on a smartphone [19–22]. Wu and Strickland-Hughes [23] conceptualize motivation as the ‘fuel’ for novel skill learning: people must have the motivation to begin learning a skill (e.g., a new control system) and then be motivated to invest the time needed to learn that skill (e.g., play a game that uses that new control system). In this paper, we focus on gaining insight into what factors influence participant’s motivation to invest time into learning a new control system.

In the last decade, gamification has received considerable attention as a tool for increasing motivation. Gamification involves adding game-like features (e.g., rewards, competition, narrative) to an otherwise mundane task to make it more enjoyable and compelling for the user [24]. In a systematic review, Lumsden, Edwards [25] concluded that gamified tasks were highly engaging and boosted participant motivation in cognitive training tasks, which can be considered similar to the sensorimotor training required to become proficient at using a new control system. In subsequent work, however, they found that participants who engaged in gamified versions of a task did not choose to engage with the task for a longer period of time [26]. One consideration is that gamification is often universally applied to all participants and thus does not consider individual differences. This approach does not incorporate ideas common to many contemporary theories of motivation which highlight the importance of an individual’s perceived competence [27], and often implicate the importance of balancing an individual’s competence (often referred to as ‘skill’) with the task difficulty [e.g., 28]. Indeed, mainstream motivation theories consistently indicate that intrinsically motivating activities should provide optimal challenges to participants to be sufficiently appealing [e.g., 29–31], an idea that has been supported by experimental studies [e.g., 32,33].

The idea of balancing challenge and failure – which is common to many theories of motivation – has been elegantly formulated in terms of success frequency [34]. Under this formulation, one would expect an inverted-U shape between motivation and success frequencies. When a participant is consistently successful (i.e., success frequency ~1) or unsuccessful (i.e., success frequency ~0) on a given task, they experience low motivation. In contrast, when they experience a mixture of success and failure (i.e., success frequency ~0.5), they are highly motivated. Several studies have implied that this mid-range success frequency is optimally motivating. Atkinson [35] showed that students who were informed that the success frequency in their experimental group was 0.3 or 0.5 compared to 0.05 or 0.75 were most motivated. Meanwhile, van der Kooij, In ‘t Veld [34] found an optimal success frequency of 0.63 in an online game. In these studies, success frequencies were either fictional [35] or left to naturally emerge [34] rather than being determined according to the participant’s current skill level.

Adaptive training is the notion of modifying the task difficulty based on the user’s current skill [36]. This approach allows researchers to keep users at the optimal challenge point which is thought to increase their motivation [28,29,31]. Adaptive training has been used extensively in rehabilitation settings (see [37] for a systematic review of virtual reality studies) where the goal is to restore motor function. Kluft, Smeets [38] compared the play duration of older adults on an exergame when success frequency was fixed at an average of 0.71 through adaptive training or fixed at 1. There was no evidence for a difference in participant’s play duration in these two conditions, but they did complete more trials in the adaptive training condition. The authors interpret this as indicating that participants were motivated to put more effort into the exergame [38].

In this paper we developed a novel control system that participants were unfamiliar with. Participants were seated on a pressure mat which recorded their movements in real-time. Their task was to learn how to adjust their body movements to control the movements of a virtual robot presented on a computer screen. The development of this control system is targeted towards 1) Individuals with limited dexterity in the hands who cannot effectively utilise touchpad or joystick controls [39,40]; 2) Teleoperators who need to use their hands for dexterous tasks such as object manipulation and thus would benefit from another input device (i.e., the pressure sensing technology) for navigating the robot; 3) Rehabilitation settings where patients are working on improving balance and core stability.

Previous work in rehabilitation settings has shown that a similar pressure sensing system can improve the sitting ability of children with cerebral palsy [41] and a Nintendo Wii exercise programme can reduce ankle muscle spasticity and improve standing balance in young people with cerebral palsy [42]. A systematic review of interactive computer play interventions in cerebral palsy (which includes several pressure based systems) highlighted the significant time investment (ranging from 5 consecutive days to 20 weeks in duration) that is needed to observe improvement in motor function [43]. This shows that there is promise in using pressure sensing technology as a control system but also highlights the need to better understanding user’s motivation to engage with such novel systems.

Experiment 1 evaluated whether participants could learn the novel control system. We also measured subjective motivation and enjoyment throughout the course of the experiment. We found that participants’ performance with the control system improved but their motivation decreased as the experiment progressed. Their performance had not plateaued (i.e., success frequency of 1) indicating that they were not yet proficient users of the control system. The noted loss of motivation is a key finding because it is a limiting factor in the goal of achieving proficiency. Experiment 2 therefore investigated the efficacy of adaptive training as a method to enhance motivation, indexed by the time participants chose to continue playing the game.

## Experiment 1

In Experiment 1, we investigated whether participants could learn to use the novel control system and evaluated their subjective motivation and enjoyment throughout the experiment.

### Methods experiment 1

#### Participants.

Thirty-nine young adult participants either volunteered to take part in the experiment or took part in return for course credit. Participants reported having no motor impairments and normal or corrected-to-normal vision. The data of two participants was lost due to technical difficulties, leaving 37 participants for analysis. None of these participants had ever used the control system before. All participants provided written informed consent. Both experiments were approved by the School of Psychology ethics committee (F1500) at the University of Nottingham and conducted in accordance with the Declaration of Helsinki. Data collection for this experiment started on 26/01/24 and ended on 12/02/24.

#### Experiment set-up.

**Fig 1:**
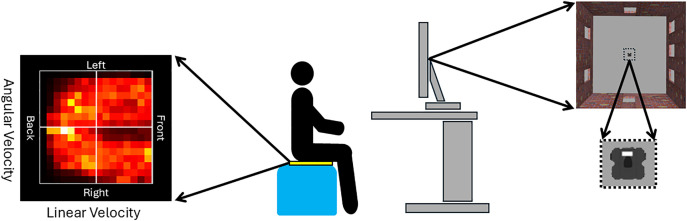
Schematic showing the experiment set-up. Participants were seated on the pressure mat (yellow) which was attached to a chair (blue). The pressure mat consisted of an array of 16 * 16 pressure sensors which is depicted by the example heat map (black indicates no pressure; white indicates maximum pressure). The linear velocity of the robot avatar was determined by the distribution of pressure in the front and back halves of the array. The angular velocity was determined by the distribution of pressure in the left and right halves of the array. The computer screen displayed the virtual environment. The robot moved around a room and had a white rectangle on it to indicate its front.

Fig 1 shows a schematic of the experiment set-up. Participants were seated on a chair on which a Sensing Tex Seating Mat (https://www.sensingmat.cloud/) consisting of an array of 16 * 16 piezoresistive pressure sensors was attached. The data from each sensor was transmitted through a USB port (5 Hz) and used to control the movements of a virtual robot presented on a computer screen. Each array of the pressure sensor data was processed and transformed into a set of movement variables to control the robot’s movement (Fig 1). The linear and angular velocity of the robot were set at a constant of 2.16 cm/s and 78 degrees/s, respectively. The direction of the robot (forwards or backwards) was dependent upon whether the sum of pressure was greater in the front (forward) or back (backwards) half of the array. The rotation of the robot (clockwise or anticlockwise) was dependent upon whether the sum of the pressure was greater in the right (clockwise) or left (anticlockwise) half of the array. When the difference in the sum of pressure was below a threshold determined based on the demographics of each individual participant, the robot did not move to ensure participants could stop the robot when desired. The linear and angular velocities were sent to the robot avatar (using twist messages, Robot Operating System, 2018) resulting in the corresponding robot movement on the computer screen.

A simulated environment (example video available on the Open Science Framework:https://osf.io/z8enb/overview) was created using the open-source robotics simulator Gazebo [44] and Robot Operating System Noetic Ninjemys (ROS). This enabled us to simulate realistic movements of a virtual robot in a controlled environment on a ThinkVision 23 inch monitor with a resolution of 1920 * 1080 pixels and refresh rate of 60 Hz. The simulated robot (~1 x 1 cm) was presented in an empty room (20 x 21 cm) with brick walls (Fig 1). Participants task was to “collect” red target spheres (1.5 cm diameter) as quickly as possible. Targets were deemed as “collected” if any part of the robot was within its boundary. Targets were only presented for a fixed duration such that participants were either successful (indicated by a positive sounding tone) or unsuccessful (indicated by a negative sounding tone) on any given trial dependent upon whether they had successfully “collected” the target in time. Once the fixed duration for a given trial had elapsed, a new target appeared 7.5 cm away from the robot’s current position. This meant that the position of targets was different across all participants because it was dependent upon the position of the robot at the time the new target was drawn.

#### Procedure.

Participants first completed a calibration procedure to capture their range of movement. This was used to determine the threshold needed to trigger the robot’s movement. Participants then completed a familiarization period to capture their baseline performance. Participants had to collect 10 targets as quickly as possible. We calculated the mean time for collecting a target by averaging their time for collecting each of the 10 targets and used that to determine the trial duration for the experimental trials so that this was personalised to account for each participant’s baseline performance. Participants then completed 80 experimental trials, split into 8 blocks (10 target collections per block). On any given trial, the target was presented for a pre-determined fixed trial duration. This allowed us to provide auditory binary feedback (success or failure) to participants on each trial and calculate their success frequency in each block as an indicator of their skill level. After each block of trials 10 trials, we asked participants to answer two questions taken from the Quick Motivation Index (QMI, van der Kooij et al, 2019): 1) How much did you enjoy the game until now and 2) How motivated are you to continue? Note that these questions require participants to engage in retrospective reporting about enjoyment and prospective reporting about their motivation. Participants were asked to respond with a value between 1 (not at all) and 10 (very much). The experiment was completed in a single session lasting approximately one hour including the explanation and reading and signing the informed consent.

### Results experiment 1

**Fig 2:**
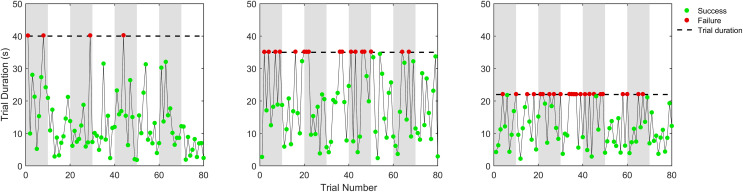
Example data from three participants showing the response time and success (coloured data points: green for success and red for failure) as a function of trial number. Each participant had a different experimental trial duration based on their baseline performance, indicated by the horizontal black dotted line. All three participants showed improved performance, indicated by more successful trials (green circles) as the experiment progressed.

The data for both experiments is available on the Open Science Framework (https://osf.io/z8enb/overview).There were individual differences in baseline performance and thus the trial duration used for the experimental trials of different participants (Fig 2). The trial duration was kept constant during the experiment (dashed horizontal lines, Fig 2) which meant that participants who learnt the task gradually experienced more successful feedback as the experiment progressed (more green dots in trials 70–80 than in trials 1–10, Fig 2).

**Fig 3:**
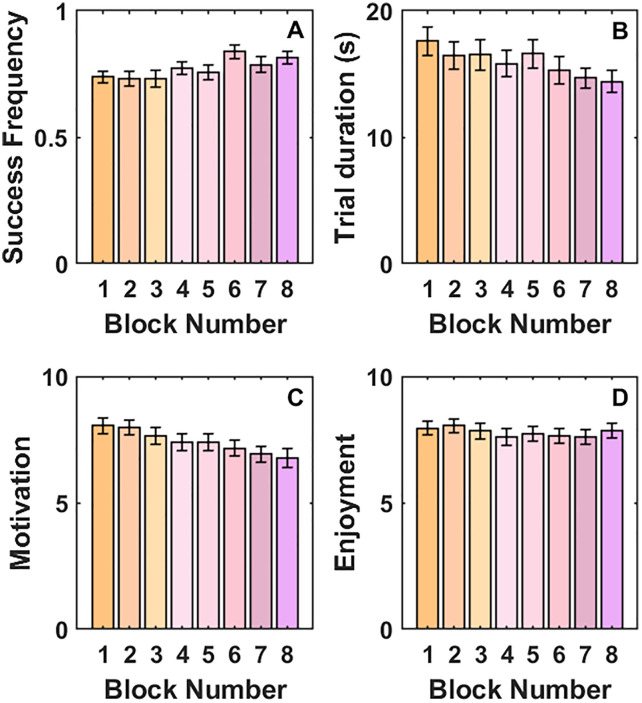
Mean (A) success frequency, (B) trial duration, (C) motivation, and (D) enjoyment as a function of Block Number. Error bars show standard error of the mean.

Fig 3 shows the mean success frequency, trial duration, motivation, and enjoyment as a function of block number. We compared participants’ success frequency, motivation, and enjoyment in Block 1 and Block 8 of the experiment. A Wilcoxon signed-rank test showed that participants had a higher success frequency in Block 8 compared with Block 1, indicative of learning (*Z* = −2.46, *p* = .014). A Wilcoxon signed-rank test showed that participant’s motivation decreased from Block 1 to Block 8 (*Z* = 3.50, *p* < .001). There was no change in enjoyment (*Z* = 0.86, *p* = .389). This shows that participants got better at using the novel control system but lost motivation relatively quickly before reaching optimal performance (i.e., a success frequency of 1).

## Experiment 2

Experiment 2 investigated whether adapting the difficulty of the task to account for participant’s current performance would increase their motivation. Our goal was to keep participants at an ‘optimal’ success frequency of between 0.6–0.8. This is in line with van der Kooij, In ‘t Veld [34] conceptualisation of this being optimally motivating because it mimics a situation where the participant’s skill and the task difficulty are approximately in balance. We compared this with two fixed difficulty conditions – a low and high success frequency – and predicted that participants in the optimal success frequency would choose to play the game for longer.

### Methods experiment 2

#### Participants.

Fifty-four young adult participants either volunteered to take part in the experiment or took part in return for course credit. Participants reported having no motor impairments and normal or corrected-to-normal vision. None of these participants had taken part in Experiment 1 and none had ever used the control system before. All participants provided written informed consent. Data collection for this experiment started on 18/01/24 and ended on 20/06/24.

#### Set-up and procedure.

The experiment set-up was identical to those used in Experiment 1, apart from the angular velocity of the robot being set to 63 degrees/s. Participants were told that their goal was to collect the targets as quickly as possible. In a baseline phase, participants collected 10 targets. The mean time it took to collect each of the 10 targets was used to determine either the fixed (low and high success frequency conditions) or starting (optimal success frequency conditions) trial duration. Participants were told that the study aimed to understand how people learn a novel control system such that they did not know that the experiment was really about motivation and how long they chose to continue playing the game for to avoid biasing their behaviour. They were told that they could decide to stop the task at any time when they were no longer motivated to continue and we used participants’ play duration as a proxy for motivation. Note this proxy differs from explicitly asking participants to self-report their motivation in Experiment 1. The maximum play duration was 18 minutes due to our recruitment method through specified timeslots of 30 minutes.

#### Design.

A between-subject design was used, with success frequency as the independent variable. Participants were randomly assigned to one of three success frequency groups: 1) low success frequency; 2) optimal success frequency; 3) high success frequency (N = 18 in each group). In the low and high success frequency groups, a fixed trial duration was used in the experimental phase. This was the participant’s baseline time + 25% of that time (high success) or baseline time – 25% of that time (low success). In the optimal success frequency condition, an adaptive procedure was used in which the trial duration was adapted based on the participant’s success frequency over the last five trials. When success frequency was < 0.4, the trial duration increased by 2 seconds. When success frequency was > 0.8, trial duration decreased by 2 seconds. Otherwise, it remained constant. The experiment was completed in a single session lasting approximately 30 minutes, including the explanation and reading and signing the informed consent.

### Results experiment 2

**Fig 4:**
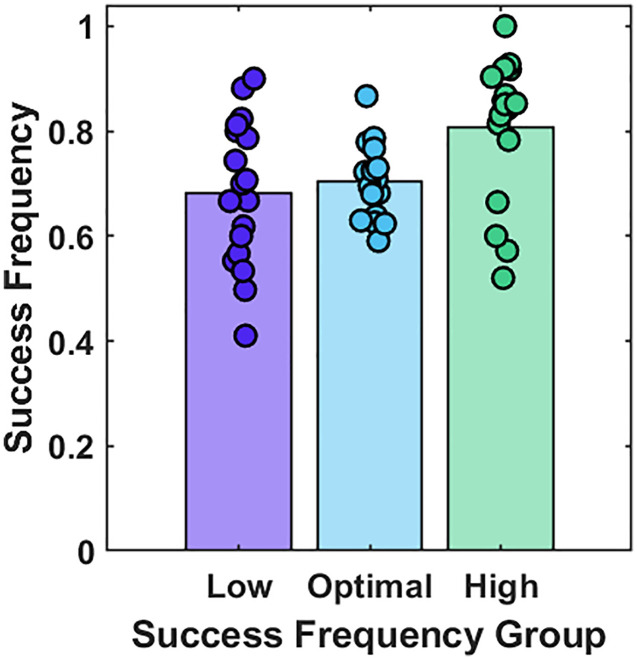
Success frequency in the three Success Frequency Groups. Bars show the mean; Coloured dots show the data for individual participants.

**Fig 5:**
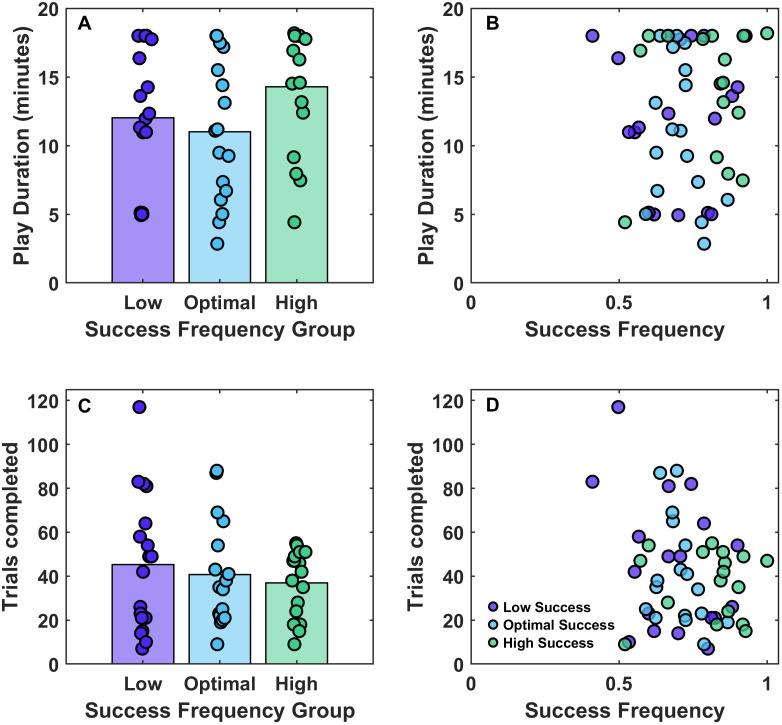
Play duration as a function of (A) experimental condition and (B) success frequency. Trials completed as a function of (C) experimental condition and (D) success frequency. Each data point is an individual participant, colour-coded according to the success frequency group that they were in.

We calculated each participant’s mean success frequency, considering a five-trial window because this is the method we used to adapt the difficulty in the optimal difficulty condition (using mean success frequency across the whole experiment did not change the qualitative pattern of results.). Two participants’ data (one each from the medium and high success frequency conditions) were removed as outliers (values more than 2 SDs from the mean). A one-way ANOVA showed that the group allocation had a significant effect on success frequency, *F*(2, 51) = 5.39, *p* = .008 (Fig 5). Post-hoc comparisons using the Tukey test showed that participants in the high success frequency condition (*M* = 0.81, *SD* = 0.14) were more successful than those in the optimal success frequency condition (*M* = 0.70, *SD* = 0.07) (*p* = .041). There was a small numerical trend in the expected direction in that participants in the optimal success frequency condition were more successful than those in the low success frequency condition (*M* = 0.68, *SD* = 0.14), but this was not significant (*p* = .840). There was considerable variability across participants’ success frequencies within the low and high success frequency conditions (coloured individual data points, Fig 4) indicating that our attempt to keep participants at specific success frequencies was not entirely successful. There was less variability in the optimal success frequency, with almost all participants falling in the desired success frequency range of 0.6–0.8, suggesting that our adaptive difficulty logic was successful.

A one-way ANOVA showed no difference in the play duration across the three groups, *F*(2, 51) = 1.96, *p* = .152 (Fig 5A). Since we did observe a range of success frequencies (0.41–1), we collated the data from all participants and conducted a quadratic regression analysis to assess whether there was an inverted-U relationship between success frequency and play duration (Play Duration ~ b1 + b2* success Frequency + b3 * (success frequency^2)). The quadratic regression showed no relation between the play duration and success frequency (b2 = −53.68, SE = 50.74, t = −1.06, p = 0.295, b3 = 38.76, SE = 35.10, t = 1.10, p = .275, as shown in Fig 5B. Fig 5D also shows no relationship between success frequency and the total number of trials completed, although this should be interpreted with caution because the number of trials completed is confounded with the trial duration which was experimentally manipulated.

## General discussion

One approach to promote learning of a novel control system is to motivate users to persist with learning, rather than attempting to accelerate the learning. In two experiments, we introduced participants to a new control system whereby they had to learn to adjust their movements whilst seated on a pressure sensing mat to control the movements of a virtual robot. Experiment 1 showed that participants could learn this control system, evidenced by an increase in success frequencies, and faster trial completions as the experiment progressed. This improvement was accompanied by a decrease in subjective motivation suggesting that identifying methods for maintaining user motivation are needed.

Experiment 2 evaluated whether adaptive training whereby the task difficulty was adjusted to account for the participants’ changing skill level would encourage participants to engage with the game for longer. We assigned participants to either a low, optimal, or high success frequency condition. There was a trend in the planned direction in that participants were most successful in the high success condition, and least successful in the low success condition. However, the actual success frequencies did not fall in the desired ranges and there was considerable variability in success frequencies across the full participant sample, ranging from 0.4 to 1. We therefore collated the data from all participants but found no evidence for an inverted-U relationship between success frequency and play duration: participants who experienced the optimal success frequency did not choose to play the game for longer. These findings do not fit with previous reports of an inverted-U relationship between success frequency and enjoyment [32] or play duration [34], which have been used as indicators of motivation. More broadly, it does not fit with the idea that intermediate challenge is optimally motivating [e.g., 27, 29, 33].

There are several factors that could explain the differences between the findings of Experiment 2 and previous research. An important distinction between [32] and our experiments is that, in [32], participants played video games against a competitor such that their skill was always evaluated relative to someone else. In our experiment, there was no external competition such that competence was always relative to oneself which may be less motivating than the extrinsic motivation inherent to competitive scenarios [32]. Meanwhile, van der Kooij, In ‘t Veld [34] studied engagement with a task that did not require *learning* a new skill because the control method was using standard keyboard presses to control an avatar. It is thus possible that, when tasked with learning something completely new, higher levels of reward are needed for participants to believe they have the potential to learn. Another consideration is that our task was less gamified than the *Speed Slice* [32] and *Covid antidote* [34] games used in previous work but we note that gamification alone has been ineffective at increasing the time that participants spend engaging with a task [26]. Experiment 2 suggests that just keeping participants at an *optimal* success frequency did not motivate them to continue playing the game for longer. A combined approach of using gamification to boost early engagement and adaptive training to continuously calibrate the game to an individual’s skill level could yield more promising results.

We observed extensive individual differences in participants’ ability to use the control system when they first started playing the game ranging from needing 7–89 seconds to collect a single target in Experiment 2 (S1 Fig). These differences were not related to neither participants’ height nor their weight (S2 Fig). It is possible that participants evaluated their skill level based on both the feedback provided and how long they felt it took them to collect a target (i.e., their perception of their skill level). Participants who found the task difficult to begin with would have experienced long trial durations such that, although they may have received positive feedback on a given trial, it may have taken them a very long time to complete that trial, so they did not perceive themselves to be skilful at the task. Clearly, evaluation of one’s skill level can be based on numerous factors and the discrepancy between different indicators of skill (i.e., successful feedback on a trial vs actual time on a trial) could explain our null finding [29–31].

Although the simplicity of formulating the balance of skill and difficulty in terms of success frequencies [34] is attractive, we propose that this formulation requires a greater understanding of how users evaluate their performance to be effective. In our experiments, participants received feedback about their performance, but it is unclear how they evaluated it. People can successfully monitor and adapt their performance on a trial-by-trial basis [45] but it is unclear whether participants are aware of their rolling performance over the course of a more continuous task (e.g., over a number of trials or minutes). It also remains unclear what trial history is considered when a learner is evaluating their skill [46]. We used a 5-trial moving window based on pilot experiments, but it is possible that participants consider either a much longer trial history (e.g., van der Kooij, In ‘t Veld [34] used 10 trials and Kluft, Smeets [38] used 9 trials) or only the previous trial. Further research is needed to understand how people evaluate their skill level on the basis of binary reward feedback. Since there were extensive individual differences on this task, this control system is a good candidate to explore this. This could be evaluated through the use of think-aloud procedures, similarly to previous research aimed at understand the demands of learning to use a powered wheelchair [47].

Adaptive training has emerged as a successful method for improving performance in settings such as rehabilitation [48] and medical image perception [49]. In these scenarios, there are clear performance related goals for the learner to achieve, namely restoration of motor function and improved diagnosis. Attempting to motivate users to utilise novel technology is a different scenario because the advantages are often less self-relevant, and slower to occur. In addition, the convenience sampling approach we used to recruit participants resulted in a narrow demographic and thus our results cannot be generalised to other populations. For future work it would be more useful to explore use-cases that were more relevant and meaningful to the participant might impact their motivation.

Our attempt to keep participants at low success frequencies was not successful. We propose that this is because we did not factor in the learning rate of participants which meant that, over time, participants got faster at the task, but the trial duration remained fixed and calibrated to the baseline period. Nevertheless, 15/17 participants in the optimal success frequency condition experienced the desired range of 0.6–0.8. Of these participants, there was a large range in the observed play durations from 3–18 minutes (Fig 5A) supporting the use of this measure as a good proxy for motivation (i.e., not all participants just carried on until the experimenter told them to stop). This finding is also indicative of clear individual differences in the extent to which the adaptive logic was motivating. This suggests that ‘optimal challenge’ – when operationalised in terms of success frequencies – is not optimally motivating for all participants based on our proxy of the time participants were willing to play the game.

Overall, we found evidence that participants could learn to use this novel control system, shown by an overall increase in success frequencies and decrease in the time to complete a trial in Experiment 1. Specifically, participants could learn to adjust their weight distribution to successfully guide a virtual robot, and this did not depend on neither the height nor weight of the participant (S2 Fig). This control system therefore holds promise for introduction into various settings (e.g., rehabilitation, teleoperation) as a method of remote control. There were, however, extensive individual differences in the starting ability of participants (S1 Fig). Such variability was not anticipated and contributed to our difficulty in keeping participants at the desired success frequencies. Understanding what demographics or characteristics makes an individual good at learning novel control systems warrants further investigation. A limitation of these experiments is that we did not record the data from the pressure mat and therefore cannot comment on the optimal movement strategies for proficient control of the robot, or how these strategies changed during the course of learning.

## Conclusion

Experiment 1 showed that participants could learn to adjust their body movements to control the movements of a virtual robot. Participants’ motivation decreased as the experiment progressed highlighting the need to consider ways to motivate learners to persist with engaging with new control systems until they reach proficiency. Experiment 2 found that adapting the difficulty of a game in line with participants’ skill level did not result in them choosing to play the game for longer than participants who played a fixed difficulty game. We conclude that pressure sensing technology has potential as a method of remote control, but further work is needed to develop interventions for motivating learners to continue engaging with learning new technology until they reach proficiency.

## Supporting information

S1 Fig Range of trial durations in Experiment 2. (PDF)

S2 Fig Relationship between participant demographics and success frequency. (PDF)
