# Supplementary material for: Pressure sensing technology for remote control: Can we motivate users to stay on the learning curve?
Source: PLoS One. 2026 Mar 10;21(3):e0340667. doi: 10.1371/journal.pone.0340667 (PMC12974823; doi:10.1371/journal.pone.0340667)
Supplement: S1 Fig — (PDF) [file pone.0340667.s001.pdf]

S1 Figure shows the trial duration(s) used for each participant in Experiment 2. There are clear individual differences in the starting trial duration (Trial 1 on the figures) ranging from 7 to 89 seconds across the whole participant sample. The range of starting durations across participants within the low, optimal, and high success frequency groups were 74, 82, and 49 seconds, respectively. This highlights that, whilst some participants found the control system easy to, others had considerable difficulty. We did not anticipate such variability and believe this contributed to the difficulty in experimentally manipulating the success frequency that a given participant experienced.

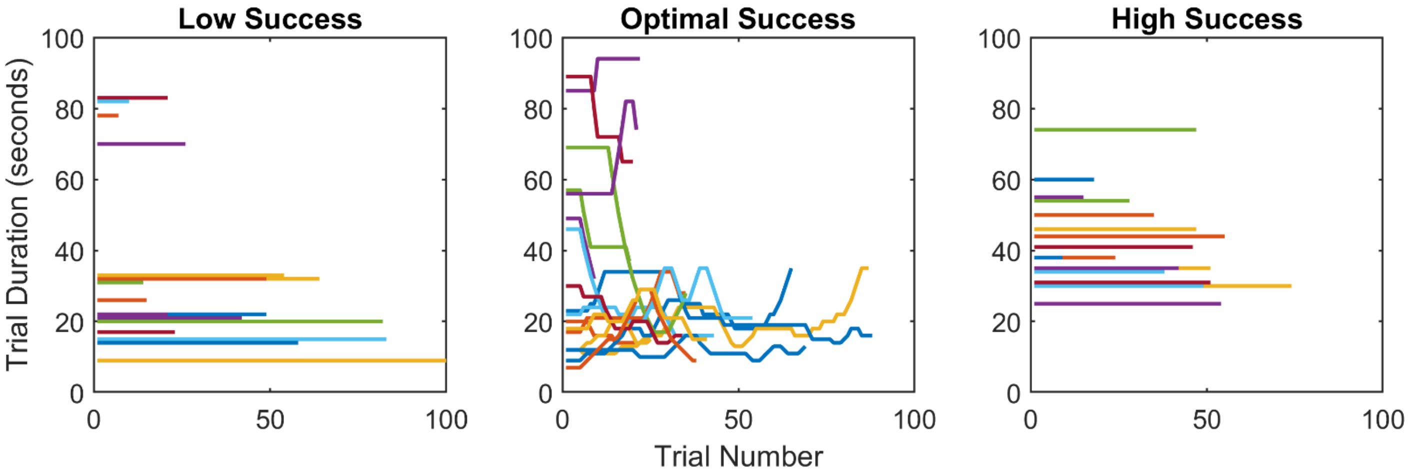

S1 Figure. Trial duration as a function of trial number for each experimental group in Experiment 2. A fixed trial duration was used in the Low and High Success conditions; an adaptive trial duration was used in the Optimal Success condition. Colours show individual participants.
