# Supplementary material for: Pressure sensing technology for remote control: Can we motivate users to stay on the learning curve?
Source: PLoS One. 2026 Mar 10;21(3):e0340667. doi: 10.1371/journal.pone.0340667 (PMC12974823; doi:10.1371/journal.pone.0340667)
Supplement: S2 Fig — (PDF) [file pone.0340667.s002.pdf]

During development of the control system and various outreach events we noted that some demographics (e.g., children) found the control system harder to use. We therefore recorded each participant's height and weight to explore any systematic relationship. S2 Figure shows that there was no relationship between participant's height and weight and success frequency in neither Experiment 1 nor Experiment 2.

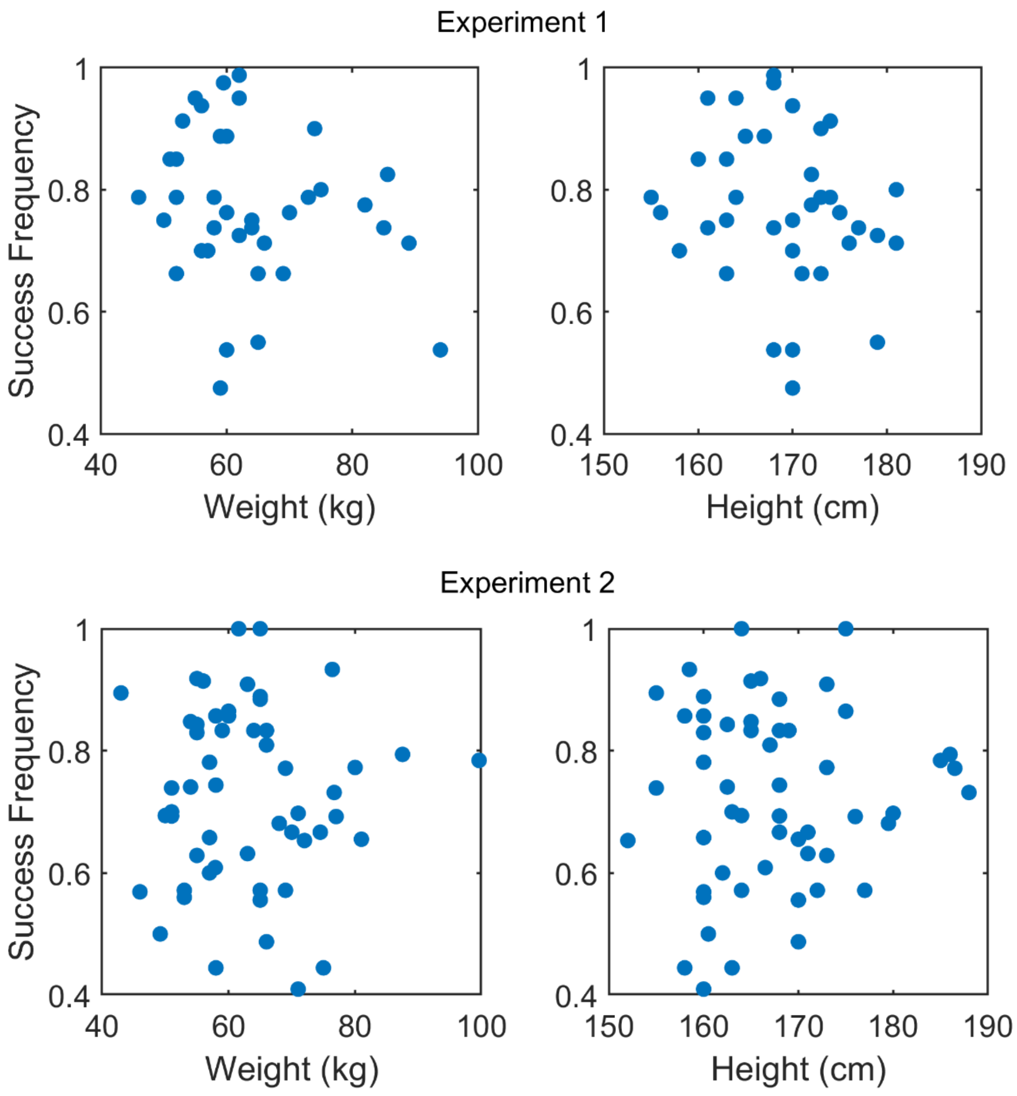

S2 Figure. Participant's weight (left panel) and height (right panel) plotted against their success frequency in Experiment 1 (top panels) and Experiment 2 (bottom panels). Note Experiment 2 aimed to experimentally manipulate success frequency so should be incorporated with caution.
